# A binuclear vanadium oxyfluoride: di-μ-oxido-bis­[fluoridooxido(1,10-phenanthro­line)vanadium(V)]

**DOI:** 10.1107/S1600536810037232

**Published:** 2010-09-25

**Authors:** Paul DeBurgomaster, Jon Zubieta

**Affiliations:** a Department of Chemistry, Syracuse University, Syracuse, New York 13244, USA

## Abstract

The title compound, [V_2_F_2_O_4_(C_12_H_8_N_2_)_2_], is a centrosymmetric binuclear vanadium(V) species with the metal ions in a distorted octa­hedral environment. The symmetry-equivalent V^V^ atoms exhibit coordination geometries defined by *cis*-terminal fluoride and oxide groups, unsymmetrically bridging oxide groups and the *N*-atom donors of the phenanthroline ligands. The crystal packing is stabilized by weak inter­molecular C—H⋯O and C—H⋯F hydrogen bonds.

## Related literature

For the properties and applications of oxyfluorido­molybdates and -­vanadates, see: Adil *et al.* (2010 ▶); Burkholder & Zubieta (2004 ▶); DeBurgomaster & Zubieta (2010 ▶); Jones *et al.* (2010 ▶); Michailovski *et al.* (2006 ▶, 2009 ▶). For examples of solid phase vanadium oxyfluorides in the presence of coligands, see: Ouellette *et al.* (2005 ▶, 2006 ▶, 2007 ▶); Ouellette & Zubieta (2007 ▶). For hydro­thermal preparation of metal oxyfluorides, see: Gopalakrishnan (1995 ▶); Whittingham (1996 ▶); Zubieta (2003 ▶).
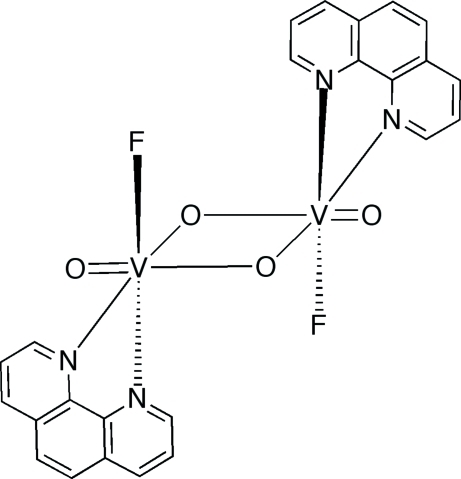


## Experimental

### 

#### Crystal data

[V_2_F_2_O_4_(C_12_H_8_N_2_)_2_]
*M* *_r_* = 564.29
Triclinic, 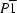

*a* = 7.8320 (9) Å
*b* = 8.4937 (10) Å
*c* = 9.2007 (11) Å
α = 113.741 (3)°
β = 102.834 (2)°
γ = 97.848 (2)°
*V* = 528.46 (11) Å^3^
*Z* = 1
Mo *K*α radiation
μ = 0.95 mm^−1^
*T* = 90 K
0.36 × 0.31 × 0.12 mm

#### Data collection

Bruker APEX CCD diffractometer
Absorption correction: multi-scan (*SADABS*; Bruker, 1998 ▶) *T*_min_ = 0.626, *T*_max_ = 0.747
5297 measured reflections
2550 independent reflections
2502 reflections with *I* > 2σ(*I*)
*R*_int_ = 0.018

#### Refinement

*R*[*F* ^2^ > 2σ(*F* ^2^)] = 0.034
*wR*(*F* ^2^) = 0.084
*S* = 1.14
2550 reflections
163 parameters
H-atom parameters constrained
Δρ_max_ = 0.41 e Å^−3^
Δρ_min_ = −0.35 e Å^−3^

### 

Data collection: *SMART* (Bruker, 1998 ▶); cell refinement: *SAINT* (Bruker, 1998 ▶); data reduction: *SAINT*; program(s) used to solve structure: *SHELXS97* (Sheldrick, 2008 ▶); program(s) used to refine structure: *SHELXL97* (Sheldrick, 2008 ▶); molecular graphics: *CrystalMaker* (Palmer, 2006 ▶); software used to prepare material for publication: *SHELXTL* (Sheldrick, 2008 ▶).

## Supplementary Material

Crystal structure: contains datablocks I, global. DOI: 10.1107/S1600536810037232/lh5133sup1.cif

Structure factors: contains datablocks I. DOI: 10.1107/S1600536810037232/lh5133Isup2.hkl

Additional supplementary materials:  crystallographic information; 3D view; checkCIF report

## Figures and Tables

**Table 1:** Hydrogen-bond geometry (Å, °)

*D*—H⋯*A*	*D*—H	H⋯*A*	*D*⋯*A*	*D*—H⋯*A*
C2—H2⋯F1^i^	0.95	2.49	3.393 (2)	160
C3—H3⋯O1^ii^	0.95	2.44	3.191 (2)	136
C6—H6⋯O1^ii^	0.95	2.46	3.200 (2)	135
C10—H10⋯O2^iii^	0.95	2.39	3.282 (2)	157

Symmetry codes: (i) 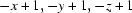; (ii) 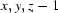; (iii) 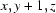.

**Fig. 1.**
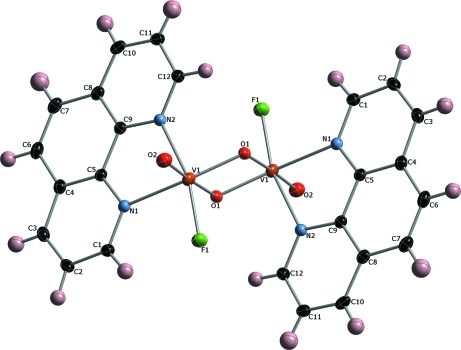
View of the molecular dimer of the title compound, with the atom-labeling scheme and the displacement ellipsoids drawn at the 50% probability level. Color scheme: vanadium, orange; oxygen, red; fluorine, green; nitrogen, light blue; carbon, black; hydrogen, pink.

**Fig. 2.**
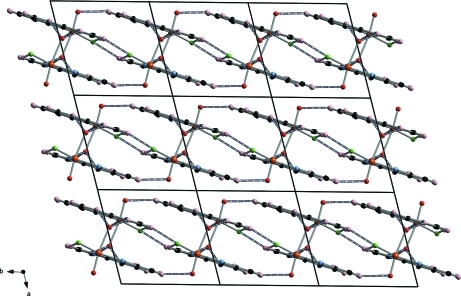
A packing diagram illustrating the C-H···O and C-H···F contacts (illustrated as multiband cylinders). Color code as for Fig. 1.

## supplementary crystallographic information

### Comment

Metal oxyfluorides exhibit a range of compositions and considerable structural versatility that give rise to useful physical properties and potential applications (Adil, *et al.*, 2010; Burkholder & Zubieta, 2004; DeBurgomaster & Zubieta, 2010; Jones *et al.*, 2010; Michailovski, *et al.*, 2006,2009; Ouellette *et al.*, 2005,2006,2007); Ouellette & Zubieta, 2007). Hydrothermal chemistry offers one approach to the preparation of novel metal oxyfluorides where the complexity of the synthetic domain allows incorporation of fluoride into metal oxide frameworks, providing unusual and often unprecedented structures (Gopalakrishnan, 1995; Whittingham, 1996; Zubieta, 2003) Furthermore, the metal-oxyfluoride core can be stabilized or modified by the introduction of appropriate coligands, such as organonitrogen donors of the pyridyl family. In the course of our investigations of the hydrothermal chemistry of metal oxides in the presence of fluoride anion, the title compound [V_2_F_2_O_4_(phen)_2_] was isolated. The compound crystallizes in the triclinic space group P1 with one binuclear molecule per unit cell, whose halves are related by a center of symmetry at the mid-point of the V···V vector. The V atoms exhibit distorted octahedral geometry with {VFO_3_N_2_} coordination (Fig. 1). The µ-bis-oxo bridging mode is characterized by a {V_2_O_2_} rhombus with alternating short-long V—O bond distances of 1.724 (1) Å and 2.316 (1) Å, respectively. The terminal oxo-groups lie in the plane of the {V_2_O_2_} rhombus and exhibit a pronounced *trans*-influence as shown by the elongated bridging oxo-group-vanadium distance, V1—O1. The coordination geometry at the vanadium sites also exhibits a fluoride ligand with V—F of 1.787 (1) Å with the V—F vector approximately normal to the {V_2_O_2_} rhombus. The V—F vectors of the binuclear unit adopt an anti-orientation with respect to the plane if the {V_2_O_2_} rhombus. The geometry is completed by the nitrogen donors of the phenanthroline ligand, which occupy positions *trans* to the short V—O bond of the rhombus and *trans* to the terminal fluoride ligand. The crystal packing is stabilized by weak intermolecular C—H···O and C—H···F hydrogen bonds which produces two-dimensional connectivity in the *bc* plane (Figure 2).

### Experimental

A mixture of V_2_O_5_ (0.062 g, 0.34 mmol), 1,10-phenanthroline (0.367 g, 2.04 mmol), H_2_O (5 ml, 277.5 mmol) and HF (0.200 ml, 5.80 mmol) in the mole ratio 1.00:6.03:1620:17.06 was stirred briefly before heating to 170 oC for 48 h (initial and final pH values of 2.5 and 2.0, respectively). Yellow rods suitable for X-ray diffraction were isolated in 65% yield. Anal. Calcd. for C_24_H_20_F_2_N_4_O_4_V_2_: C, 51.0; H, 2.84; N, 9.92; F, 6.73. Found: C, 50.7;H,3.01;N, 9.67; F, 6.55.

### Refinement

All hydrogen atoms were discernable in the difference Fourier map. The hydrogen atoms were placed in calculated positions with C—H = 0.95 Å and included in the riding model approximation with *U*_iso_(H) = 1.2*U*_eq_(C).

### Crystal data

**Table:**

[V_2_F_2_O_4_(C_12_H_8_N_2_)_2_]	*Z* = 1
*M**_r_* = 564.29	*F*(000) = 284.0
Triclinic, *P*1	*D*_x_ = 1.773 Mg m^−^^3^*D*_m_ = 1.77 (2) Mg m^−^^3^*D*_m_ measured by flotation
Hall symbol: -P 1	Mo *K*α radiation, λ = 0.71073 Å
*a* = 7.8320 (9) Å	Cell parameters from 3266 reflections
*b* = 8.4937 (10) Å	θ = 2.4–28.3°
*c* = 9.2007 (11) Å	µ = 0.95 mm^−^^1^
α = 113.741 (3)°	*T* = 90 K
β = 102.834 (2)°	Plate, yellow
γ = 97.848 (2)°	0.36 × 0.31 × 0.12 mm
*V* = 528.46 (11) Å^3^	

### Data collection

**Table:**

Bruker APEX CCD diffractometer	2550 independent reflections
Radiation source: fine-focus sealed tube	2502 reflections with *I* > 2σ(*I*)
graphite	*R*_int_ = 0.018
Detector resolution: 512 pixels mm^-1^	θ_max_ = 28.1°, θ_min_ = 2.5°
φ and ω scans	*h* = −10→9
Absorption correction: multi-scan (*SADABS*; Bruker, 1998)	*k* = −11→11
*T*_min_ = 0.626, *T*_max_ = 0.747	*l* = −12→12
5297 measured reflections	

### Refinement

**Table:**

Refinement on *F*^2^	Primary atom site location: structure-invariant direct methods
Least-squares matrix: full	Secondary atom site location: difference Fourier map
*R*[*F*^2^ > 2σ(*F*^2^)] = 0.034	Hydrogen site location: inferred from neighbouring sites
*wR*(*F*^2^) = 0.084	H-atom parameters constrained
*S* = 1.14	*w* = 1/[σ^2^(*F*_o_^2^) + (0.0297*P*)^2^ + 0.608*P*] where *P* = (*F*_o_^2^ + 2*F*_c_^2^)/3
2550 reflections	(Δ/σ)_max_ = 0.001
163 parameters	Δρ_max_ = 0.41 e Å^−^^3^
0 restraints	Δρ_min_ = −0.35 e Å^−^^3^

### Special details

**Table:**

Geometry. All e.s.d.'s (except the e.s.d. in the dihedral angle between two l.s. planes) are estimated using the full covariance matrix. The cell e.s.d.'s are taken into account individually in the estimation of e.s.d.'s in distances, angles and torsion angles; correlations between e.s.d.'s in cell parameters are only used when they are defined by crystal symmetry. An approximate (isotropic) treatment of cell e.s.d.'s is used for estimating e.s.d.'s involving l.s. planes.
Refinement. Refinement of *F*^2^ against ALL reflections. The weighted *R*-factor *wR* and goodness of fit *S* are based on *F*^2^, conventional *R*-factors *R* are based on *F*, with *F* set to zero for negative *F*^2^. The threshold expression of *F*^2^ > σ(*F*^2^) is used only for calculating *R*-factors(gt) *etc*. and is not relevant to the choice of reflections for refinement. *R*-factors based on *F*^2^ are statistically about twice as large as those based on *F*, and *R*- factors based on ALL data will be even larger.

### Fractional atomic coordinates and isotropic or equivalent isotropic displacement parameters (Å^2^)

**Table:**

	*x*	*y*	*z*	*U*_iso_*/*U*_eq_	
V1	0.32341 (4)	0.84898 (4)	0.86984 (4)	0.01413 (10)	
F1	0.43262 (16)	0.67119 (15)	0.83110 (14)	0.0199 (2)	
O1	0.39833 (17)	0.97396 (17)	1.08350 (15)	0.0130 (3)	
O2	0.11464 (19)	0.74797 (18)	0.82733 (18)	0.0182 (3)	
N1	0.3194 (2)	0.8039 (2)	0.61559 (19)	0.0128 (3)	
N2	0.2439 (2)	1.0719 (2)	0.83968 (19)	0.0131 (3)	
C1	0.3604 (2)	0.6671 (2)	0.5064 (2)	0.0152 (4)	
H1	0.3896	0.5771	0.5364	0.018*	
C2	0.3623 (3)	0.6503 (3)	0.3487 (2)	0.0168 (4)	
H2	0.3938	0.5514	0.2746	0.020*	
C3	0.3182 (2)	0.7782 (3)	0.3023 (2)	0.0161 (4)	
H3	0.3184	0.7682	0.1957	0.019*	
C4	0.2724 (2)	0.9249 (2)	0.4151 (2)	0.0143 (3)	
C5	0.2775 (2)	0.9315 (2)	0.5709 (2)	0.0121 (3)	
C6	0.2197 (2)	1.0641 (3)	0.3789 (2)	0.0169 (4)	
H6	0.2146	1.0606	0.2735	0.020*	
C7	0.1770 (2)	1.2007 (3)	0.4937 (2)	0.0167 (4)	
H7	0.1410	1.2904	0.4666	0.020*	
C8	0.1852 (2)	1.2119 (2)	0.6554 (2)	0.0144 (3)	
C9	0.2355 (2)	1.0768 (2)	0.6925 (2)	0.0126 (3)	
C10	0.1417 (2)	1.3488 (2)	0.7797 (2)	0.0168 (4)	
H10	0.1096	1.4451	0.7621	0.020*	
C11	0.1464 (3)	1.3406 (3)	0.9264 (2)	0.0180 (4)	
H11	0.1145	1.4302	1.0103	0.022*	
C12	0.1982 (3)	1.2007 (2)	0.9531 (2)	0.0159 (4)	
H12	0.2008	1.1976	1.0556	0.019*	

### Atomic displacement parameters (Å^2^)

**Table:**

	*U*^11^	*U*^22^	*U*^33^	*U*^12^	*U*^13^	*U*^23^
V1	0.01424 (17)	0.01694 (17)	0.01513 (17)	0.00489 (12)	0.00478 (12)	0.01048 (13)
F1	0.0238 (6)	0.0184 (5)	0.0203 (6)	0.0093 (5)	0.0074 (5)	0.0096 (5)
O1	0.0156 (6)	0.0145 (6)	0.0113 (6)	0.0054 (5)	0.0052 (5)	0.0070 (5)
O2	0.0174 (7)	0.0177 (6)	0.0218 (7)	0.0049 (5)	0.0070 (5)	0.0103 (6)
N1	0.0115 (7)	0.0145 (7)	0.0125 (7)	0.0039 (6)	0.0032 (6)	0.0060 (6)
N2	0.0123 (7)	0.0144 (7)	0.0120 (7)	0.0044 (6)	0.0033 (6)	0.0054 (6)
C1	0.0142 (8)	0.0153 (8)	0.0155 (9)	0.0053 (7)	0.0042 (7)	0.0059 (7)
C2	0.0153 (9)	0.0181 (9)	0.0121 (8)	0.0032 (7)	0.0049 (7)	0.0021 (7)
C3	0.0136 (8)	0.0210 (9)	0.0115 (8)	0.0022 (7)	0.0043 (7)	0.0059 (7)
C4	0.0100 (8)	0.0177 (8)	0.0139 (8)	0.0009 (6)	0.0023 (6)	0.0075 (7)
C5	0.0097 (8)	0.0137 (8)	0.0129 (8)	0.0028 (6)	0.0028 (6)	0.0064 (7)
C6	0.0129 (8)	0.0230 (9)	0.0171 (9)	0.0011 (7)	0.0024 (7)	0.0134 (8)
C7	0.0136 (8)	0.0182 (9)	0.0213 (9)	0.0024 (7)	0.0030 (7)	0.0134 (8)
C8	0.0098 (8)	0.0151 (8)	0.0176 (9)	0.0018 (6)	0.0017 (7)	0.0083 (7)
C9	0.0099 (8)	0.0137 (8)	0.0136 (8)	0.0025 (6)	0.0026 (6)	0.0061 (7)
C10	0.0123 (8)	0.0131 (8)	0.0231 (10)	0.0035 (7)	0.0015 (7)	0.0081 (7)
C11	0.0162 (9)	0.0154 (9)	0.0174 (9)	0.0067 (7)	0.0023 (7)	0.0031 (7)
C12	0.0159 (9)	0.0168 (8)	0.0132 (8)	0.0061 (7)	0.0038 (7)	0.0046 (7)

### Geometric parameters (Å, °)

**Table:**

V1—O2	1.6203 (14)	C3—H3	0.9500
V1—O1	1.7241 (13)	C4—C5	1.404 (2)
V1—F1	1.7871 (12)	C4—C6	1.438 (3)
V1—N2	2.1724 (16)	C5—C9	1.430 (2)
V1—N1	2.2052 (16)	C6—C7	1.360 (3)
V1—O1^i^	2.3162 (13)	C6—H6	0.9500
N1—C1	1.330 (2)	C7—C8	1.438 (3)
N1—C5	1.358 (2)	C7—H7	0.9500
N2—C12	1.329 (2)	C8—C9	1.402 (2)
N2—C9	1.359 (2)	C8—C10	1.409 (3)
C1—C2	1.403 (3)	C10—C11	1.373 (3)
C1—H1	0.9500	C10—H10	0.9500
C2—C3	1.376 (3)	C11—C12	1.400 (3)
C2—H2	0.9500	C11—H11	0.9500
C3—C4	1.415 (3)	C12—H12	0.9500
			
O2—V1—O1	104.98 (7)	C2—C3—H3	120.4
O2—V1—F1	102.29 (6)	C4—C3—H3	120.4
O1—V1—F1	104.75 (6)	C5—C4—C3	116.98 (17)
O2—V1—N2	91.64 (6)	C5—C4—C6	118.79 (17)
O1—V1—N2	90.52 (6)	C3—C4—C6	124.22 (17)
F1—V1—N2	155.64 (6)	N1—C5—C4	123.54 (17)
O2—V1—N1	98.29 (6)	N1—C5—C9	116.19 (16)
O1—V1—N1	152.31 (6)	C4—C5—C9	120.27 (16)
F1—V1—N1	84.36 (6)	C7—C6—C4	120.81 (17)
N2—V1—N1	73.81 (6)	C7—C6—H6	119.6
O2—V1—O1^i^	170.39 (6)	C4—C6—H6	119.6
O1—V1—O1^i^	76.95 (6)	C6—C7—C8	121.25 (17)
F1—V1—O1^i^	86.10 (5)	C6—C7—H7	119.4
N2—V1—O1^i^	78.88 (5)	C8—C7—H7	119.4
N1—V1—O1^i^	77.68 (5)	C9—C8—C10	117.11 (17)
V1—O1—V1^i^	103.05 (6)	C9—C8—C7	118.67 (17)
C1—N1—C5	118.04 (16)	C10—C8—C7	124.21 (17)
C1—N1—V1	125.58 (12)	N2—C9—C8	123.62 (17)
C5—N1—V1	116.33 (12)	N2—C9—C5	116.17 (16)
C12—N2—C9	117.95 (16)	C8—C9—C5	120.20 (17)
C12—N2—V1	124.57 (13)	C11—C10—C8	118.87 (17)
C9—N2—V1	117.45 (12)	C11—C10—H10	120.6
N1—C1—C2	122.64 (17)	C8—C10—H10	120.6
N1—C1—H1	118.7	C10—C11—C12	120.26 (18)
C2—C1—H1	118.7	C10—C11—H11	119.9
C3—C2—C1	119.50 (17)	C12—C11—H11	119.9
C3—C2—H2	120.2	N2—C12—C11	122.16 (18)
C1—C2—H2	120.2	N2—C12—H12	118.9
C2—C3—C4	119.27 (17)	C11—C12—H12	118.9
			
O2—V1—O1—V1^i^	−170.30 (6)	C1—N1—C5—C4	1.1 (3)
F1—V1—O1—V1^i^	82.35 (6)	V1—N1—C5—C4	178.74 (13)
N2—V1—O1—V1^i^	−78.46 (6)	C1—N1—C5—C9	−179.58 (16)
N1—V1—O1—V1^i^	−24.09 (15)	V1—N1—C5—C9	−1.9 (2)
O1^i^—V1—O1—V1^i^	0.0	C3—C4—C5—N1	−1.6 (3)
O2—V1—N1—C1	−91.43 (15)	C6—C4—C5—N1	177.65 (16)
O1—V1—N1—C1	121.45 (17)	C3—C4—C5—C9	179.14 (16)
F1—V1—N1—C1	10.21 (15)	C6—C4—C5—C9	−1.7 (3)
N2—V1—N1—C1	179.27 (16)	C5—C4—C6—C7	0.6 (3)
O1^i^—V1—N1—C1	97.44 (15)	C3—C4—C6—C7	179.75 (17)
O2—V1—N1—C5	91.11 (13)	C4—C6—C7—C8	0.8 (3)
O1—V1—N1—C5	−56.00 (19)	C6—C7—C8—C9	−1.1 (3)
F1—V1—N1—C5	−167.25 (13)	C6—C7—C8—C10	−179.54 (18)
N2—V1—N1—C5	1.81 (12)	C12—N2—C9—C8	1.4 (3)
O1^i^—V1—N1—C5	−80.02 (12)	V1—N2—C9—C8	179.58 (13)
O2—V1—N2—C12	78.41 (16)	C12—N2—C9—C5	−177.17 (16)
O1—V1—N2—C12	−26.60 (15)	V1—N2—C9—C5	1.0 (2)
F1—V1—N2—C12	−156.19 (15)	C10—C8—C9—N2	0.1 (3)
N1—V1—N2—C12	176.56 (16)	C7—C8—C9—N2	−178.51 (16)
O1^i^—V1—N2—C12	−103.19 (15)	C10—C8—C9—C5	178.59 (16)
O2—V1—N2—C9	−99.64 (13)	C7—C8—C9—C5	0.0 (3)
O1—V1—N2—C9	155.36 (13)	N1—C5—C9—N2	0.6 (2)
F1—V1—N2—C9	25.8 (2)	C4—C5—C9—N2	179.98 (16)
N1—V1—N2—C9	−1.48 (12)	N1—C5—C9—C8	−178.00 (16)
O1^i^—V1—N2—C9	78.77 (13)	C4—C5—C9—C8	1.4 (3)
C5—N1—C1—C2	0.1 (3)	C9—C8—C10—C11	−1.6 (3)
V1—N1—C1—C2	−177.28 (13)	C7—C8—C10—C11	176.93 (17)
N1—C1—C2—C3	−0.8 (3)	C8—C10—C11—C12	1.6 (3)
C1—C2—C3—C4	0.3 (3)	C9—N2—C12—C11	−1.4 (3)
C2—C3—C4—C5	0.8 (3)	V1—N2—C12—C11	−179.42 (14)
C2—C3—C4—C6	−178.35 (17)	C10—C11—C12—N2	−0.1 (3)

Symmetry codes: (i) −*x*+1, −*y*+2, −*z*+2.

### Hydrogen-bond geometry (Å, °)

**Table:**

*D*—H···*A*	*D*—H	H···*A*	*D*···*A*	*D*—H···*A*
C2—H2···F1^ii^	0.95	2.49	3.393 (2)	160
C3—H3···O1^iii^	0.95	2.44	3.191 (2)	136
C6—H6···O1^iii^	0.95	2.46	3.200 (2)	135
C10—H10···O2^iv^	0.95	2.39	3.282 (2)	157

Symmetry codes: (ii) −*x*+1, −*y*+1, −*z*+1; (iii) *x*, *y*, *z*−1; (iv) *x*, *y*+1, *z*.
